# Caloric beverage consumption patterns in Mexican children

**DOI:** 10.1186/1475-2891-9-47

**Published:** 2010-10-21

**Authors:** Simon Barquera, Fabricio Campirano, Anabelle Bonvecchio, Lucia Hernández-Barrera, Juan A Rivera, Barry M Popkin

**Affiliations:** 1 Nutrition and Health Research Center, National Institute of Public Health, Av. Universidad 655, Col. Sta. Ma. Ahuacatitlan, Cuernavaca, Mor. CP 62508, Mexico; 2 Department of Nutrition, School of Public Health and the Carolina Population Center, University of North Carolina, 123 W. Franklin St., Chapel Hill, NC 27516, USA

## Abstract

**Background:**

Mexico has seen a very steep increase in child obesity level. Little is known about caloric beverage intake in this country as well as all other countries outside a few high income countries. This study examines overall patterns and trends in all caloric beverages from two nationally representative surveys from Mexico.

**Methods:**

The two nationally representative dietary intake surveys (1999 and 2006) from Mexico are used to study caloric beverage intake in 17, 215 children. The volume (ml) and caloric energy (kcal) contributed by all beverages consumed by the sample subjects were measured. Results are weighted to be nationally representative.

**Results:**

The trends from the dietary intake surveys showed very large increases in caloric beverages among pre-school and school children. The contribution of whole milk and sugar-sweetened juices was an important finding. Mexican pre-school children consumed 27.8% of their energy from caloric beverages in 2006 and school children consumed 20.7% of their energy from caloric beverages during the same time. The three major categories of beverage intake are whole milk, fruit juice with various sugar and water combinations and carbonated and noncarbonated sugared-beverages.

**Conclusion:**

The Mexican government, greatly concerned about obesity, has identified the large increase in caloric beverages from whole milk, juices and soft drinks as a key target and is initiating major changes to address this problem. They have already used the data to shift 20 million persons in their welfare and feeding programs from whole to 1.5% fat milk and in a year will shift to nonfat milk. They are using these data to revise school beverage policies and national regulations and taxation policies related to an array of less healthful caloric beverages.

## Introduction

During the last two decades a very large increase in obesity has been documented in Mexico using nationally representative surveys.[1,2] Increases have been observed across all age groups. The prevalence of obesity and overweight increased by 39.7% in school age children from 1999 to 2006.[2] The highest increase in child obesity and overweight prevalence is observed during primary school. When children enter primary school at 6-y of age their average prevalence of obesity and overweight is 24.3%. However, at 12-y of age when they are graduating, their prevalence is 32.5%, reflecting a 12.2 percentage point increase. This increase is probably a result of several lifestyle factors. However, diverse preliminary surveys and research identified a proliferation of caloric beverages and large increases in these products as being widespread in Mexican families, along with the lack of available, potable water in many schools in Mexico.[3] Mexico is the second largest consumer of soft drinks in the world [4] and also consumes extensive sugar-sweetened flavored waters termed "agua frescas" The proportion of families purchasing soft drinks has increased over the years, as well as the per capita milliliters consumed.[4] Some studies suggest that type 2 diabetes mellitus is increasing in Mexican children and adolescents.[5,6] Also, the association between obesity and diverse cardiovascular risk factors in adults has been documented.[6-8]

Diverse lifestyle conditions have been related to the rise in obesity and Nutrition Related Noncommunicable Chronic Diseases (NR-NCDs) in Mexican children. Some of the main determinants include lack of physical activity, hours of TV watching, low quality and energy dense diets, and high caloric sweetener intake.[3] Caloric beverages have been recognized as an important source of energy in children and have been associated with increased risk of overweight.[9,10] Whole milk is widely consumed in the country and subsidized to low income children.[11] Juices are equally prominent in the diet. This article focuses on the caloric beverage component of the Mexican diet.

There is a large literature on the role of whole milk and milk in general in child weight gain and health in general. The most recent review from the US Dietary Guidelines Committee notes that "Moderate evidence suggests that there is not a relationship between intake of calcium and/or dairy (milk and milk products) and adiposity in children and adolescents."[12] The American Academy of Pediatrics and the American Heart Association have strongly endorsed this approach of providing only skim milk to preschoolers aged 2 and older.[13,14] One of the best sets of studies come from Finland in a random trial in which one set of infants were fed low-fat diets from age 7 months onwards while the other set consumed whole milk. These studies found that low-saturated-fat dietary intervention from infancy until 5 years of age safely and effectively reduced serum cholesterol concentration. At seven years, this intervention favorably influenced not only the serum total and LDL cholesterol concentrations but also the LDL particle size in boys. And by nine years, this intervention had a positive effect on insulin resistance index in 9-year-old children.[15,16]

While there is a growing consensus that there is a lack of *adequate energy compensation *for modifications of energy intakes via fluids, we do not understand the exact mechanisms--other than all beverages have a weak satiety value.[17-20] The work of Mattes and others shows that low satiety and lack of energy compensation is linked with caloric beverages. This has spurred scholars to look at the overall beverage intake patterns and their healthfulness.[21,22] As shown in several meta-analyses and reviews of the role of sugar-sweetened beverages (SSBs), increased intake is associated with higher energy intake, weight gain, obesity, and diabetes.[23-25] Most of this research has focused on the effects of soft drinks and fruit drinks. There are fewer studies that found that fruit juice has the same effect.[26,27] Other metabolic effects are much less frequently studied.[28-30]

Although child obesity has been recognized as a national priority, very little is known in Mexico about the factors that could effectively determine and modify this condition and its important health risks. The objective of this study is to describe the characteristics and trends in caloric beverages in pre-school and school children in Mexico using nationally representative data from the 1999 Mexican Nutrition Survey and the 2006 Mexican Health and Nutrition Survey.

## Methods

### Sample design

We analyzed the Mexican Nutrition Survey 1999 (MNS-99) and the Mexican Health and Nutrition Survey 2006 (MHNS-06). Both the MNS-99 and the MHNS-06 was national, cross-sectional, multi-stage, stratified surveys representative of the country. The MNS-99 was conducted between October 1998 and March 1999. The MHNS-06 was conducted between October 2005 and May 2006. Both surveys had sampling power to disaggregate into urban (population ≥2,500 inhabitants) and rural (population <2,500 inhabitants) areas. The objective of these surveys was to characterize the nutritional status as well as the food and nutrient patterns of the Mexican population. Due to budget restrictions, the MNS-99 only collected information of children and women of reproductive age (12-49y), while the MHNS-06 collected information regarding children, as well as both adult men and women of all ages (≥19y). A detailed description of the sampling procedures and survey methodology has been published elsewhere.[2,31] A total of 1204 and 3552 preschool children (1-4y) were evaluated in 1999 and 2006 respectively. In school age children (5-11y) a total of 2496 and 8716 were evaluated in 1999 and 2006 respectively. Children without complete dietary records were dropped from the analysis presented here.

The MNS-99 and the MHNS-06 samples were drawn to be representative of four regional strata, North, Central, Mexico City and South. The four regional strata, with common geographic and socio economic characteristics, were (1) North: Baja California, Southern Baja California, Coahuila, Durango, Nuevo Leon, Sonora, Sinaloa, Tamaulipas and Zacatecas, (2) Central: Aguascalientes, Colima, Guanajuato, Hidalgo, Jalisco, Mexico, Michoacan, Nayarit, Querétaro, San Luis Potosí and Tlaxcala, (3) Mexico City and (4) South: Campeche, Chiapas, Guerrero, Morelos, Oaxaca, Puebla, Quintana Roo, Tabasco, Veracruz and Yucatan. This regionalization scheme has been used in diverse epidemiologic transition analysis for within country comparisons.[32,33]

Informed consent was obtained from each subject or subject's parent or guardian for their participation in the study. The survey protocol was approved by the Ethics Committee of the National Institute of Public Health, Mexico.

### Dietary intake data

Elsewhere there are more detailed descriptions of the dietary data from the 1999 and 2006 surveys.[34,35] The methods are different in the two surveys and this has been addressed in depth in a companion paper.[35] In 1999, a single 24-hour dietary recall was utilized [36] from women and children. In 2006, a previously validated semi quantitative Food Frequency Questionnaire (FFQ) [37] including 101 foods and 14 food groups was used, this questionnaire included the 95% most consumed foods reported in the 24-h dietary recalls collected in the MNS-99. In both cases for children under 12, parents and/or caretakers were involved in handling the surveys with assistance from the children when useful. Standardized personnel applied the FFQ to a nationally-representative sub-sample. Respondents were queried on their dietary intake over the previous 7 days, including the portion size. From these food items a total of 17 beverages were recorded, 13 sweetened and unsweetened beverages (such as tea, coffee, juice, and unique local beverages made with grains, fruit juice and sugar), and four types of milk. This instrument collected information of food intake for the previous seven days in a sub-sample of approximately 16,100 households and all individuals in the household. This sub-sample was representative of the country, country regions, urban and rural areas, and states. Dietitians collected both sets of data and converted food consumption data into grams or milliliters.

Intakes and percentages of energy, carbohydrates, proteins, and fat adequacies reported on the individual level greater than 5 standard deviations from their respective means were excluded from the analysis. Likewise, energy adequacy percentages less than 25% were eliminated, as they were implausible values. Dietary data from 3,959 children in preschool children were obtained of which 407 observations were excluded (10.3%). In school children dietary data were collected from 9,383 and 667 observations (7.1%) were excluded from the analysis. Due to the data elimination during the cleaning process, we calculated new sampling weights.

### Socio-economic measures

A socio-economic status (SES) index has been developed in Mexico using a combination of household conditions (i.e., flooring material, ceiling, walls, and number of persons residing in the household), basic services infrastructure (i.e., water source and disposal), and possession of domestic appliances (i.e., radio, television and refrigerator).[38-40] This approach was used to create a comparable SES index for the 1999 and 2006 surveys.

### Beverage classification

Our grouping of beverages that follows was utilized by other scholars.[41] This grouping placed beverages into categories based on caloric intake and overall potential health benefits. Thus we created the following categories: a) High energy (soft drinks, sweetened tea and coffee, sweetened juice and fruit drinks, and atole--a typical Mexican beverage made with sugar, corn meal usually, or other substitutes such as rice flour and corn starch) b) High energy some benefit (whole milk, milk flavored, fruit juice), c) Low energy (sweetened diet beverages, unsweetened tea and coffee) and d) water. At the time of the MHNS-06, only trivial amounts of reduced fat milk was consumed (in the MNS-99 basically no reduced fat milk was available on the market).

### Estimation of beverage intake

Energy intake was estimated using a comprehensive nutrient composition database compiled from diverse references. Major outliers were reviewed case by case and corrected when possible.[42-46] Using the MNS-99 and MHNS-06 we generated measures of percent calories from beverages, proportion of beverages that include some type of caloric sweetener by age, gender, urban or rural locations, geographic areas and socio-economic status index tertile. Consumption patterns were examined using a variety of characterizations, including a) the dichotomous characterization as consumed beverage for the proportion who consume each type of beverage; b) per capita consumption of each beverage for the age-gender grouping [in milliliters (ml) and kilocalories (kcal)]; c) the amount of beverages consumed by each consumer of these beverages (in ml and kcal); and the percent contribution to total energy intake (among consumers and per-capita). To describe consumption (ml and kcal), arithmetic means were calculated. Differences across energy intake by socio-demographic factors were evaluated using ANOVA with Tukey's adjustment for multiple comparisons (termed in STATA linear combination estimators). Significant differences between consumption trends between 1999 and 2006 were evaluated using a T-test for independent samples; a p-value < 0.05 was considered significant. All calculations were weighted by expansion factors and adjusted for the complex sampling survey design using the STATA 9.0 SVY module for complex surveys.[47]

## Results

### Trends in consumption 1999-2006

**Figure 1:**
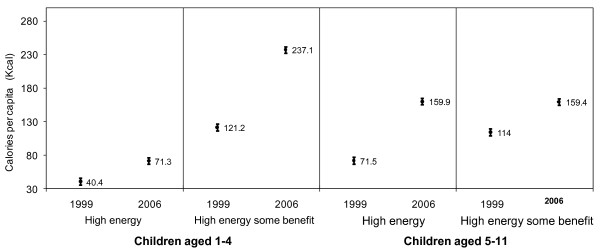
**Beverage Consumption Trends of Mexican Children, 1999-2006**. * Statistically significant difference between 1999 and 2006 at P < 0.01 Note: High energy (soft drinks, sweetened tea and coffee, sweetened juice and fruit drinks, and atole); High energy some benefit (whole milk, milk flavored, fruit juice), and Low energy (sweetened diet beverages, unsweetened tea and coffee. Data weighted to be nationally representative. Total energy intake = Children aged 1-4; In 1999 = 949; In 2006 = 1070 kcal (median) Children aged 5-11; In 1999 = 1377; In 2006 = 1501 kcal (median)

Consumption of caloric beverages was analyzed by beverage group for pre-school and school children. At the national level, the per capita calories from beverages in pre-school children increased from 161 kcal in 1999 to 310 kcal in 2006 (p < 0.05). Similarly, in school-age children, consumption increased from 185 kcal to 323 kcal per capita from 1999 to 2006 (p < 0.05) (Figure 1).

### Detailed consumption patterns in 2006

Pre-school children consumed approximately 794 ml of beverages per day, including plain water in 2006. These beverages contributed to 27.8% of the total reported energy intake. From all beverage types in this age group, the "high sugar some benefit" group, which included mainly whole milk, provided the largest number and proportion of calories (21% of the total estimated daily energy intake) (Table 1).

**Table 1:** Daily beverage Consumption of 1-4 year old Mexican Children, 2006*

	n	Consumers (%)	Per capita consumption (ml) Mean (SE)	Amount per consumer (ml) Mean (SE)	Calories per capita ( kcal) Mean (SE)	Calories per consumer (kcal) Mean (SE)	**Contribution from the consumed calories per capita to TEI % (95%IC)**^**‡**^	Contribution from the consumed calories to TEI % (95%IC)
**High Energy**								
Regular Soft drinks	2376	68.0	40.5 (1.7)	59.5 (2.3)	18.3 (0.78)	26.9 (1.03)	1.7 (1.5-1.8)	2.4 ((2.2-2.7)
Sweetened Coffee & Tea	1389	37.5	27.3 (1.4)	72.7 (2.9)	5.5 (0.27)	14.6 (0.59)	0.6 (0.5-0.7)	1.6 (1.4-1.7)
Sweetened beverages^§^	2260	65.2	72.3 (2.7)	110.9 (3.5)	38.1 (1.4)	58.4 (1.9)	3.3 (3.1-3.6)	5.1 (4.8-5.5)
Atole	469	10.9	8.8 (0.81)	80.4 (5.2)	9.4 (0.87)	86.3 (5.5)	0.9 (0.7-1.12)	8.6 (7.3-9.9)
Group	3326	94.9	148.9 (3.5)	156.9 (3.6)	71.3 (1.8)	75.0 (1.9)	6.6 (6.2-6.9)	6.9 (6.5-7.3)
**High Energy -Some benefit**								
Whole milk	2789	79.9	366.1 (8.3)	458.4 (8.3)	220.0 (5.0)	275.4 (5.0)	19.7 (18.8-20.6)	24.5 (23.7-25.6)
Whole flavored	172	5.4	11.1 (1.8)	205.7 (22.8)	10.8 (1.8)	201.2 (22.3)	0.8 (0.5-1.0)	14.3 (11.4-17.2)
Unsweetened fruit juice	806	25.3	13.8 (0.92)	54.5 (2.71)	6.2 (0.41)	24.6 (1.2)	0.54 (0.47-0.62)	2.15 (1.9-2.4)
Group	2979	85.2	391.0 (8.6)	459.1 (8.6)	237.1 (5.4)	278.4 (5.5)	21.0 (20.1-21.9)	24.7 (23.7-25.6)
**Low Energy**								
Diet Soft drinks & unsweetened flavor	103	3.7	2.5 (0.5)	67.4 (10.9)	0	0.16 (0.08)	0	0.01 (0.06-0.02)
Unsweetened coffee & tea	118	3.7	3.0 (0.73)	81.0 (10.7)	0.54 (0.01)	1.45 (0.24)	0.06 (0.03-0.08)	0.15 (0.09-0.22)
Skim milk	57	0.2	5.3 (1.3)	287.3 (50.2)	2.1 (0.54)	116.9 (20.4)	0.19 (0.09-0.29)	10.4 (6.5-14.3)
Group	271	9.2	10.8 (1.6)	118.4 (13.8)	2.2 (0.54)	38.5 (9.2)	0.20 (0.10-0.30)	3.4 (1.8-5.1)
**Plain Water**	3306	93.3	243.6 (6.2)	260.9 (6.4)	--	--	--	--
**TOTAL**	**3549**	**99.9**	**794.4 (11.2)**	**794.7 (11.2)**	**310.5 (5.6)**	**311.9 (5.6)**	**27.8 (26.9-28.7)**	**27.9 (27.0-28.8)**

*Weighted data

^‡^Total energy intake = 1131.8 kcal (mean) 1070.3 (median)

^§^Includes: sweetened fruit water, sugar-added orange juice, sweetened and flavored water

School age children consumed 1254 ml per capita of beverages in 2006. These beverages contributed to 20.7% of the total energy intake. In contrast with pre-school children, consumption of high-sugar beverages was more important (10.3% of the total energy intake as opposed to only 6.6% in the younger children) (p = 0.01). The proportional role of sweetened beverages, such as fruit water, sugar-added orange juice, sweetened and flavored water, increased also (Table 2).

**Table 2:** Daily beverage Consumption of 5-11 year old Mexican Children, 2006*

	n	Consumers (%)	Per capita consumption (ml) Mean (SE)	Amount per consumer (ml) Mean (SE)	Calories per capita ( kcal) Mean (SE)	Calories per consumer (kcal) Mean (SE)	**Contribution from the consumed calories per capita to TEI % (95%IC)**^**‡**^	Contribution from the consumed calories to TEI % (95%IC)
**High Energy**								
Regular Soft drinks	6537	76.1	121.8 (3.9)	160.0 (4.7)	54.9 (1.7)	72.2 (2.1)	3.5 (3.3-3.8)	4.7 (4.4-4.9)
Sweetened Coffee & Tea	4124	44.2	80.7 (2.8)	182.4 (4.8)	16.7 (0.58)	37.8 (1.0)	1.24 (1.1-1.3)	2.8 (2.6-2.8)
Sweetened beverages^§^	5350	60.6	148.5 (4.5)	245.1 (6.1)	77.5 (2.4)	127.8 (3.4)	4.8 (4.5-5.1)	7.9 (7.5-8.3)
Atole	1051	10.5	10.0 (0.62)	95.6 (3.7)	10.7 (0.67)	102.7 (4.0)	0.78 (0.68-0.88)	7.45 (6.8-8.1)
Group	8405	95.8	361.0 (6.6)	376.8 (6.6)	159.9 (3.0)	166.9 (3.0)	10.3 (10.0-10.7)	10.8 (10.4-11.1)
**High Energy -Some benefit**								
Whole milk	6527	77.2	240.8 (5.0)	311.9 (5.4)	144.4 (3.0)	187.1 (3.3)	9.2 (8.8-9.5)	11.9 (11.5-12.3)
Whole flavored	355	4.0	5.6 (0.58)	139.6 (7.5)	5.5 (0.57)	136.5 (7.3)	0.30 (0.24-0.37)	7.62 (6.7-8.5)
Unsweetened fruit juice	1601	21.0	21.1 (1.0)	100.2 (2.9)	9.5 (0.45)	45.3 (1.3)	0.61 (0.55-0.67)	2.9 (2.7-3.1)
Group	6897	81.2	267.5 (5.2)	329.2 (5.5)	159.4 (3.1)	196.2 (3.3)	10.1 (9.7-10.4)	12.2 (12.0-12.8)
**Low Energy**								
Diet Soft drinks & unsweetened flavor	227	3.2	6.8 (1.45)	208.8 (28.8)	0	1.0 (--)	0	0.08 ---
Unsweetened coffee & tea	195	2.1	2.0 (0.28)	92.4 (6.8)	0.04 (0.001)	2.0 (0.24)	0.003 (0.002-0.004)	0.14 (0.11-0.17)
Skim milk	165	2.6	8.5 (1.6)	329.5 (3.5)	3.5 (0.65)	134.0 (14.3)	0.25 (0.16-0.35)	9.8 (7.7-11.9)
Group	563	7.6	17.3 (2.17)	226.6 (18.7)	3.5 (0.65)	75.2 (10.4)	0.26 (0.16-0.35)	5.5 (4.0-7.0)
**Plain Water**	8122	92.4	607.7 (9.0)	657.9 (8.9)	--	--	--	--
**TOTAL**	**8711**	**99.9**	**1253.6 (12.3)**	**1254.7 (12.3)**	**322.9 (5.5)**	**325.1 (4.5)**	**20.7 (20.2-21.1)**	**20.8 (20.4-21.3)**

*Weighted data

^‡^Total energy intake = 1547 kcal (mean)

^§^Includes: sweetened fruit water, sugar-added orange juice, sweetened and flavored water

School age children's milk consumption was slightly lower (144 kcal) than pre-school children's consumption (220 kcal). This is equivalent to a 34.5% lower intake (p < 0.01). A total of 607 ml of plain water was consumed per capita in school age children. In pre-school children only 243 ml was consumed.

Thus, the main change in beverage consumption for pre-school children occurred in whole milk and juice water. In school children the change in consumption of whole milk is important, but flavored waters and soft drinks changed dramatically.

Tables 3 and 4 describe consumption of energy beverages and their contribution to the diet. In pre-school children across all the socio-demographic variables whole milk was the most important caloric beverage. It contributes at least 200 kcal per capita daily with the exception of rural areas and low socio-economic status children. In contrast, children from urban areas consume 236 kcal. Children from Mexico City consume 262 kcal and those in high socio-economic status consume 259 kcal. Next in importance are the sugar-sweetened juices which contribute 29 kcals in the low socio-economic status children and 51 kcal in those from the high SES (p < 0.05). In the population from the low SES tertile, calories from coffee and tea with sugar, and atole were higher than in the highest SES tertile (p < 0.05), in rural than urban areas (p < 0.05) and south region than in the other country regions (p < 0.05). Males had a significantly higher intake compared to females from juices and sweetened beverages (p < 0.05).

**Table 3:** Per capita energy intake from beverages among Mexican Children Aged 1-4 by Socio-Demographic Factors, 2006

	National	Area		Socioeconomic Status		
		Rural	Urban	**Low**^**a**^	**Medium**^**b**^	**High**^**c**^
**Beverage**						
Regular Soft drinks	18.3 (0.78)	12.5 (0.78)^£^	20.4 (1.0)	15.2 (1.1)^b.c^	19.8 (1.5)^a^	21.3 (1.6)^a^
Sweetened Coffee & Tea	5.5 (0.27)	10.3 (0.59) ^£^	3.7 (0.28)	8.4 (0.48)^b,c^	3.8 (0.44)^a,c^	2.5 (0.38)^a,b^
Sweetened beverages^§^	38.1 (1.4)	30.2 (2.2) ^£^	41.0 (1.8)	29.2 (1.6)^b,c^	40.3 (2.5)^a,c^	50.8 (3.9)^a,b^
Atole	9.4 (0.87)	20.0 (2.2.) ^£^	5.4 (0.81)	15.5 (1.6)^b,c^	5.3 (1.2)^a^	4.4 (1.4)^a^
Whole milk	220.0 (5.0)	176.5 (7.2) ^£^	236.3 (6.3)	173.1 (6.5)^b,c^	252.0 (8.8)^a^	258.9 (11.7)^a^
Whole flavored	10.8 (1.8)	7.2 (1.8)	12.2 (2.3)	9.4 82.2)	9.0 (2.5)	15.9 (5.3)
Unsweetened fruit juice	6.2 (0.41)	4.4 (0.64) ^£^	6.9 (0.52)	4.4 (0.50)^b,c^	7.0 (0.81)^a^	8.3 (0.94)^a^
Unsweetened coffee & tea	0.054 (0.01)	0.08 (0.28)	0.04 (0.01)	0.08 (0.02) ^b^	0.02 (0.007) ^a^	0.05 (0.02)
Skim milk	2.1 (0.54)	0.44 (0.14) ^£^	2.8 (0.75)	0.40 (0.11) ^b,c^	3.6 (1.34) ^a^	3.2 (1.22) ^a^
TOTAL	**310.5 (5.6)**	**261.6 (8.4) **^**£**^	**328.9 (7.4)**	**255.9 (7.2) **^**b,c**^	**341.0 (9.8) **^**a**^	**365.3 (14.2) **^**a**^

	**Region**				**Gender**	

	North^a^	Central^b^	Mexico^c^	South^d^	Males	Females
**Beverage**						
Regular Soft drinks	27.1 (1.9)^b,c,d^	19.1 (1.6)^a,d^	17.5 (2.3)^a,d^	12.7 (0.83)^a,b.c^	17.9 (1.0)	18.7 (1.2)
Sweetened Coffee & Tea	3.0 (0.36)^b,d^	4.1 (0.42) ^a,d^	3.3 (0.70)^d^	9.3 (0.56) ^a,b.c^	5.3 (0.38)	5.6 (0.39)
Sweetened beverages^§^	40.4 (3.1)	40.3 (2.6)^d^	40.3 (5.1)	33.6 (1.9)^b^	41.2 (2.2) ^£^	34.8 (1.8)
Atole	2.3 (1.4)^d^	3.5 (0.70)^d^	3.9 (1.7)^d^	21.7 (2.2) ^a,b.c^	8.6 (1.2)	10.2 (1.3)
Whole milk	204.0 (10.5)^b,c^	236.4 (9.0)	262.5 (16.1)^d^	194.0 (7.4) ^a,b.c^	226.3 (6.8)	213.5 (7.3)
Whole flavored	10.6 (3.2)	4.3 (1.3)^c,d^	21.4 (8.4)^b^	12.0 (2.7)^b^	9.8 (2.6)	11.8 (2.4)
Unsweetened fruit juice	5.2 (0.87)^b^	7.7 (0.89)^a,b,c^	6.3 (1.2)^d^	5.4 (0.49) ^a,b.c^	5.3 (0.52) ^£^	7.2 (0.64)
Unsweetened coffee & tea	0.02 (0.01)	0.03 (0.01)	0.10 (0.05)	0.06 (0.02)	0.06 (0.02)	0.05 (0.01)
Skim milk	2.1 (1.08)	0.69 (0.47)	4.5 (2.04)	2.5 (1.1)	2.0 (0.66)	2.3(0.89)
**TOTAL**	**294.6 (11.7) **^**c**^	**316.1 (9.9)**	**359.9 (20.9) **^**a,d**^	**291.4 (9.3) **^**c**^	**316.6 (8.2)**	**304.3 (8.1)**

£ = significantly different at p < 0.05

^a,b,c,d ^= Within a subgroup, means in a row with superscripts without a common letter differ (p < 0.05).

**Table 4:** Per capita energy intake from beverages among Mexican Children Aged 5-11 by Socio-Demographic Factors, 2006

	National	Area		Socioeconomic Status		
		**Rural**	**Urban**	**Low^a^**	**Medium^b^**	**High^c^**

**Beverage**						
Regular Soft drinks	54.9 (1.7)	36.5 (1.3)^£^	62.7 (2.4)	39.9 (2.8)^b,c^	60.9 (2.4)^a,c^	72.5 (4.4)^ab^
Sweetened Coffee & Tea	16.7 (0.58)	27.7 (1.0) ^£^	12.1 (0.70)	26.6 (1.0)^b,c^	12.3 (0.93)a,c	6.0 (0.55)a,b
Sweetened beverages^§^	77.5 (2.4)	61.2 (2.4) ^£^	84.3 (3.3)	59.4 (2.2)^b,c^	85.2 (3.4)^a^	97.6 (7.5)^a^
Atole	10.7 (0.67)	19.9 (1.4) ^£^	6.9 (0.73)	17.3 (1.2)^b,c^	6.1 (0.69)^a^	5.6 (1.6)^a^
Whole milk	144.4 (3.0)	85.5 (3.0) ^£^	169.1 (4.0)	97.8 (4.4)^b,c^	167.6 (4.9)^a,c^	192.7 (7.2)^ab^
Whole flavored	5.5 (0.57)	4.9 (1.0)	5.7 (0.69)	5.2 (0.81)	6.3 (1.2)	4.7 (0.81)
Unsweetened fruit juice	9.5 (0.45)	7.0 (0.60) ^£^	10.6 (0.59)	7.0 (0.56)^b,c^	9.9 (0.81)^a,c^	13.1 (1.1)^a,b^
Unsweetened coffee & tea	0.04 (0.01)	0.05 (0.001)	0.04 (0.01)	0.03 (0.005)	0.05 (0.015)	0.04 (.0014)
Skim milk	3.5 (0.68)	1.7 (0.47) ^£^	4.2 (0.94)	0.66 (0.19) ^b,c^	3.95 (0.92) ^a^	7.8 (2.44) ^a^
**TOTAL**	**322.9 (5.5)**	**244.6 (6.4) **^£^	**355.6 (6.8)**	**254 (7.76)**^b,c^	**352.3 (7.53) **^a,c^	**400.2 (11.6) **^a,b^

	**Region**				**Gender**	

	North^a^	Central^b^	Mexico^c^	South^d^	Males^£^	Females

**High Sugar**						
Regular Soft drinks	80.9 (3.8)^b,c,d^	55.7 (3.4)^a,d^	63.0 (7.2)a,d	38.9 (1.9)^a,b,c^	58.5 (2.8) ^£^	51.4 (2.8)
Sweetened Coffee & Tea	8.4 (0.61) ^b,c,d^	10.9 (0.97) ^a,d^	14.1 (2.3) ^a,d^	28.5 (1.1) ^b,c,d^	17.1 (0.90)	16.3 (0.76)
Sweetened beverages^§^	73.4 (3.9)	82.3 (4.9)	88.2 (9.8)	71.7 (2.9)	78.9 (3.3)	76.1 (3.5)
Atole	3.0 (0.77)^b,d^	6.5 (1.0)^a,d^	6.0 (1.5)^a,b^	20.8 (1.4)^a,b,c^	10.7 (1.0)	10.7 (0.86)
Whole milk	137.2 (5.0)^b,c,d^	171.4 (5.7)^a,c,d^	205 (13.9)^a,b,d^	104.5 (3.6)^a,b,c^	157.8 (4.4) ^£^	131.4 (4.1)
Whole flavored	4.8 (1.0)	3.3 (0.78)^c,d^	10.1 (2.8)^b^	7.4 (1.1)^c^	5.8 (0.78)	5.2 (0.82)
Unsweetened fruit juice	8.3 (0.92)	9.4 (0.81)	11.0 (1.7)	10.1 (0.68)	9.5 (0.66)	9.6 (0.62)
Unsweetened coffee & tea	0.04 (0.01)	0.03 (0.005)	0.11 (0.10)	0.05 (0.01)	0.03 (0.01)	0.05 (0.01)
Skim milk	4.8 (1.3) ^d^	3.8 (1.4)	8.1 (3.6)	1.5 (0.35) ^a^	4.0 (1.2)	3.0 (0.61)
**TOTAL**	**320.9 (8.4) **^c,d^	**343.4 (10.6) **^c,d^	**406.5 (19.8) **^a,d^	**283.4 (7.1) **^a,c^	**342.4 (7.5) **^£^	**303.7 (6.2)**

£ = significantly different at p < 0.05

^a,b,c,d ^= Within a subgroup, means in a row with superscripts without a common letter differ (p < 0.05).

In school children the contribution of whole milk to total beverage caloric intake is lower than in pre-school children across all socio-demographic factors, and important differences were observed; urban children had a 58% higher consumption than rural children, and higher SES tertile children had a 57% higher consumption than those in the lower SES tertile. The contribution of soft drinks was higher in this age group compared to the pre-school children, and overall caloric consumption from beverages was higher with the exception of children from the south country region.

Children residing in the north region consumed almost double the energy from soft drinks.

## Discussion

This study describes caloric beverage consumption in Mexico using nationally representative data from two points in time. Our results suggest that caloric beverages could be playing an important role in the genesis of obesity and nutrition-related chronic diseases early in life. From the caloric beverages consumed in Mexico among children, whole milk is the one that contributes the most to total energy intake. The reduction in whole milk intake in the 5-11y age group appears to be accompanied by an increase in sugar-sweetened beverages including soft drinks and juices. A previous analysis made by our group found that price elasticities for whole milk and soft drinks are three times greater than those of all other foods. This suggests that as income increases consumption will continue to rise. This earlier study [35] also presented nationally food expenditures and other data for adolescent and adults that replicate these trends seen for preschoolers and school age children.

There are important limitations in the results presented. In particular, examining trends in calories or volume obtained from beverages between the MNS-1999 24-hour recall data and the MNHS-2006 FFQ is fraught with potential for error. Scholars often report 24-hour recalls utilized various probes in obtaining more detailed food intake data on snacks and any other eating events outside a normal meal, but they tend to underestimate the intake of food items stigmatized by society.[48] In contrast, the FFQ often overestimates dietary intake; however, more recent, rigorous analyses found FFQ's also underestimate caloric intake.[49-51] Evidence of underreporting of energy intake has been documented with the 24-hour recall and to a lesser extent with the FFQ, thus the increase in consumption over time could be overestimated slightly by the methods even though real consumption is higher.[52,53]

Without a methodological cross-over study to understand how to bridge the data between these two methods in Mexico, we are unsure if our trends over- or under-estimated the shifts during this seven-year period in beverage intake. In addition, based on limited in-depth research on child caloric beverages, we suspect that there might be an underestimation of caloric beverages consumed by school age children, particularly beverages consumed in the school (such as fruit juices and sugared flavored water). However, there is minimal research to allow us to know if there is any underestimation, and if so, by how much.

Given the amount of whole milk consumed by the children and its contribution to fat and total energy intake, it is important to communicate evidence of improvements in diet and health status by counseling shifts to low-saturated fat diets in children.[16] Also, it is important to underline that independent of the problem of obesity, the high consumption of whole milk in the pre-school and school child could be associated to the lipid profile showed by Mexican children and adults, where dyslipidemias are a national public health problem.[7,54] Clear epidemiologic and experimental evidence indicates that elevated cholesterol levels in childhood are positively associated with the risk of future coronary heart disease. Diet changes that lower fat, saturated fat and cholesterol intake in children and adolescents can be applied safely and acceptably resulting in improved plasma lipid profiles. If these diet changes continue in adult life, cardiovascular risk factors could be lessened.[55,56]

A recent workshop on preventing obesity in children and youth of Mexican origin, sponsored by the Institutes of Medicine, concluded among their recommendations at the school level that beverages offered to children and youth in and around the school environment should be regulated.[3,57] Furthermore, there is direct policy action being taken on this topic in Mexico. The Minister of Health established a Beverage Guidance Panel.[58] Based on this and related research, the Minister of Health and the Mexican Beverage.

### Mexican Government Actions

Using results from this study and a comparable one from adults, the Minister of Health organized a beverage guidance panel to review and organize a set of actions for the government to reduce energy from beverages as one component of its obesity prevention campaign for all age groups.[59] A comparable paper on adult beverage trends has been published.[35] The Minister has worked with the government to institute a number of actions already.

One action already taken has been to replace all whole milk in the feeding programs of approximately twenty million Mexicans with 1.5% milk. The government social support programs--Oportunidades, Liconsa and PAL-- distribute foods and/or provide cash transfers. Initially for a 2-month trial the program gave reduced fat milk only to senior citizens. This worked well and now all individuals receiving milk or using vouchers obtain 1.5% milk.

A study is underway in Mexico City to work out ways to provide potable water in a series of schools and use this pilot to launch this first in several states, and then nationally. This is being led by the National Institute of Public Health. Ultimately, the Secretary of Education will allow only skim milk and water in the schools. School breakfast programs are removing flavored milk and using reduced fat milk only.

First and foremost, the government is considering a range of regulations and taxation plans related to reducing added sugar in beverages.[58] In addition, these authors are involved with others in Mexico in creating a Front of the Package Labeling system that will label only beverages with limited saturated fat and added sugars with a healthy beverage label. This is an ongoing activity sponsored jointly by the Finance Ministry, the Food Standards Agency and the Ministry of Health that will be finalized late in 2009 and implemented sometime in 2010.

## Conclusion

Mexican preschoolers and school age children consume the highest documented levels of calories from beverages as a proportion of total energy intake (27.8 and 20.7% respectively). Calories from beverages increased significantly from 1999 to 2006 while energy from non-beverage food calories remained constant. Although the increase over time could be overestimated by the methods used in the 1999 and 2006 surveys, it is clear that consumption of caloric beverages is increasing. Future research needs to understand the role of the increase in caloric beverages in preschooler and child obesity.

## Abbreviations

NR-NCDs: Nutrition Related Noncommunicable Chronic Diseases; SSBs: sugar-sweetened beverages; MNS-99: Mexican Nutrition Survey 1999; MHNS-06: Mexican Health and Nutrition Survey 2006; FFQ: Food Frequency Questionnaire; SES: socio-economic status

## Conflicts of interests

The authors declare that they have no competing interests.

## Authors' contributions

SB, BMP and JAR designed the study with assistance from FC and AB. LHB undertook all the data manipulation and statistical analyses with support from FC. SB and BMP did the initial draft of the paper and all coauthors reviewed and edited the final text. All authors read and approved the final manuscript.
